# LSA-DDI: Learning Stereochemistry-Aware Drug Interactions via 3D Feature Fusion and Contrastive Cross-Attention

**DOI:** 10.3390/ijms26146799

**Published:** 2025-07-16

**Authors:** Shanshan Wang, Chen Yang, Lirong Chen

**Affiliations:** 1 School of Economics and Management, Yan’an University, Yan’an 716000, China; ustc_wangshanshan@126.com; 2 College of Computer Science, Inner Mongolia University, Hohhot 010021, China; yc_777060@163.com

**Keywords:** 3D molecular spatial representation, contrastive attention mechanism, drug–drug interaction prediction

## Abstract

Accurate prediction of drug–drug interactions (DDIs) is essential for ensuring medication safety and optimizing combination-therapy strategies. However, existing DDI models face limitations in handling interactions related to stereochemistry and precisely locating drug interaction sites. These limitations reduce the prediction accuracy for conformation-dependent interactions and the interpretability of molecular mechanisms, potentially posing risks to clinical safety. To address these challenges, we introduce LSA-DDI, a Spatial-Contrastive-Attention-Based Drug–Drug Interaction framework. Our 3D feature extraction method captures the spatial structure of molecules through three features—coordinates, distances, and angles—and fuses them to enhance the model of molecular spatial structures. Concurrently, we design and implement a Dynamic Feature Exchange (DFE) mechanism that dynamically regulates the flow of information across modalities via an attention mechanism, achieving bidirectional enhancement and semantic alignment of 2D topological and 3D spatial structure features. Additionally, we incorporate a dynamic temperature-regulated multiscale contrastive learning framework that effectively aligns multiscale features and enhances the model’s generalizability. Experiments conducted on public drug databases under both warm-start and cold-start scenarios demonstrated that LSA-DDI achieved competitive performance, with consistent improvements over existing methods.

## 1. Introduction

In recent years, combination therapy has gradually replaced single-drug treatment approaches in clinical practice for disease management. However, the concurrent use of multiple medications carries potential risks of adverse drug interactions. These interactions not only pose risks to patient health and safety, but can even become life threatening. Research data indicate that adverse drug interactions account for approximately 30% of all reported adverse drug reactions and represent a leading cause of drug withdrawals from the market [1,2,3,4,5,6]. Such issues not only significantly increase morbidity and mortality, but also lead to substantial increases in public healthcare expenditure, due to the high costs associated with drug development and increased treatment expenses.

Predicting drug–drug interactions (DDIs) presents substantial challenges, as conventional approaches primarily depend on time-intensive in vitro experiments and clinical trials—methods known for their high costs and limited efficiency [7]. Recent advances in computational approaches, particularly through machine learning [8] and deep learning [9], have revolutionized this field. These techniques synergize multidisciplinary expertise, spanning computational science, pharmaceutical chemistry, pharmacology, and bioinformatics, offering powerful tools to accelerate drug development and optimize clinical medication strategies [2,10]. A growing number of deep-learning-driven computational frameworks have emerged as efficient alternatives for DDI prediction, demonstrating remarkable effectiveness in addressing this complex problem. Consequently, DDI prediction has become a prominent research frontier in artificial intelligence applications for healthcare.

Recent years have seen rapid progress in deep learning and neural network technologies, which have demonstrated remarkable capabilities in such fields as natural language processing, video recognition, and image identification. However, these applications are primarily confined to Euclidean spaces and are not easily applicable to graph-structured data. Advances in graph neural networks have significantly shifted focus to applying deep learning to graph data, leading to progress in such tasks as link prediction and node classification. The richness of graph data in computational biology, including molecular graphs, protein–protein interaction networks, and drug–target interaction networks, has driven the widespread adoption of graph neural networks in bioinformatics.

Many recent methods for predicting drug adverse interactions use graph neural networks (GNNs) such as GCN [11], GAT [12], and GIN [13], which are widely applied in DDI tasks [1,14,15,16,17]. These methods can be grouped into two categories: molecular-graph-based and DDI-network-based. The former uses Python’s RDKit toolkit [18] to convert SMILES [19] into molecular graphs, treating atoms and bonds as nodes and edges. For example, Deac et al. [20] presented a GNN architecture for DDI prediction using drug molecular structures. Wang et al. [21] integrated genomic and pharmacological features with GCN and attention mechanisms to identify synergistic drug combinations. Zhang et al. [22] employed centrality, spatial, and edge coding with a lightweight attention mechanism to extract structural features from drug graphs. Since drug molecules can be broken down into bioactive substructures, such as functional groups, some studies have predicted DDIs by analyzing interactions between these substructures [1,23,24,25]. Nyamabo et al. [26] input molecular graph representations of drug pairs into GAT to extract substructural information. Yu et al. [27] embedded substructure information based on a predefined list and proposed a substructure-aware tensor neural network for DDI prediction. Multimodal models, which integrate diverse data types (images, text, sequences, and graphs), enhance predictive power and robustness. Fu et al. [28] proposed a hierarchical interaction network for clinical trial outcome prediction using multimodal data. Duan et al. [29] combined BERT and GNN models to extract textual and two-dimensional (2D) graph information for DDI prediction. They also used interactive attention vectors to address relationship overlap in drug interactions. Compared to 2D graph representations, three-dimensional (3D) graph representations can capture molecular spatial relationships and stereochemistry, improving prediction accuracy.

As research in the DDI-prediction field continues to advance, several innovative approaches have emerged. Recent studies have begun exploring the integration of 2D and 3D molecular information, as well as multimodal feature fusion based on attention mechanisms. For example, Molormer [30] offers a new perspective on DDI prediction by combining 2D molecular graphs with spatial information. This method primarily enhances molecular representation by fusing 2D structural features with partial 3D descriptors, thereby improving prediction performance. Meanwhile, MHCADDI (2023) employs a co-attention mechanism to integrate various drug features, significantly improving the model’s ability to capture complex relationships between drugs and offering a more effective solution for predicting interactions involving novel drugs.

However, these methods still face limitations when dealing with complex stereochemical interactions. Although Molormer [30] incorporates spatial information, it may not fully exploit the spatial structure of molecules during feature extraction. Likewise, co-attention-based fusion models such as MHCADDI [31] may struggle to accurately identify the key interaction regions of drug pairs during the feature-fusion process. In addition, some 3D-based DDI predictors often rely on simple geometric descriptors or single-scale features, which limits their ability to comprehensively capture stereochemical characteristics.

To address these challenges, the LSA-DDI model introduces significant improvements and innovations. First, LSA-DDI adopts a systematic 3D spatial encoding strategy—including coordinate, distance, and angle encoding—to comprehensively capture stereochemical information. Second, it introduces a bidirectional cross-attention module and a dynamic feature-exchange mechanism, which not only more precisely identify the critical interaction regions of drug pairs, but also achieve deep semantic alignment between 2D and 3D features. Finally, LSA-DDI incorporates a multiscale contrastive learning framework, along with a dynamic temperature-adjustment mechanism, to effectively align and integrate molecular features across levels, enabling the model to capture complex stereochemical interaction patterns at multiple scales. These innovations endow LSA-DDI with stronger representational power and higher predictive accuracy to model intricate stereochemical relationships.

We conducted a series of experiments on the DrugBank benchmark dataset to evaluate the model’s effectiveness and generalizability. In warm-start tasks, the model achieved an AUROC value exceeding 98%, demonstrating its exceptional performance. For the most challenging cold-start tasks, the model achieved competitive performance on the DrugBank dataset, showing consistent improvements compared to state-of-the-art DDI-prediction models.

Our paper makes two main contributions.

- To address the problem of insufficient 3D information, LSA-DDI first employs molecular conformation and dynamic feature exchange to align 2D and 3D feature information of drugs. Random rotation is also used to incorporate the stereoscopic structure of drug molecules, enhancing the model’s generalization of new drug prediction.
- In this study, a dynamic feature exchange framework with dynamic temperature-regulated contrastive learning precisely locates drug interaction sites and boosts the model’s generalization ability. Moreover, we assessed our method on standard benchmark tests.

The results demonstrate that our approach led to modest but consistent improvements across most evaluation metrics.

## 2. Methods

### 2.1. Problem Settings

Given a set of drugs G, an interaction type space $I=\{I_{1},I_{2},\ldots ,I_{m}\}$, and a training dataset $D={\left\{{\left(G_{i},G_{j},r\right)}_{k}\right\}}_{k=1}^{n}$ consisting of annotated drug pairs with their corresponding interaction types, we seek a sophisticated mapping function,

f:G×G×I→[0,1],

designed to predict the likelihood, $p=f(G_{i},G_{j},I_{t})$, that a given drug pair, $\left(G_{i},G_{j}\right)$, will exhibit a specific interaction type, $I_{t}$. The mapping function, *f*, captures the complex relationships between drugs and their interaction types, providing a probabilistic assessment of the interactions.

### 2.2. Theoretical Background

To provide a clearer understanding of the core techniques integrated within the LSA-DDI framework, this section outlines the foundational concepts of attention mechanisms and contrastive learning.

#### 2.2.1. Attention Mechanism

An attention mechanism, a fundamental component in many neural architectures, can be formally defined as

(1) $$\mathrm{Attention}\left(Q,K,V\right)=\mathrm{softmax}\left(\frac{QK^{⊤}}{\sqrt{d_{k}}}\right)V$$

This formulation maps a query and a set of key–value pairs to an output, where the query, keys, values, and output are all vector representations. The output is computed as a weighted sum of the values, with the weights determined by a compatibility function measuring the similarity between the query and each key. In practice, multiple queries are processed in parallel, with the queries, keys, and values represented as matrices *Q*, *K*, and *V*, respectively [32]. This mechanism enables the model to selectively attend to the most relevant parts of the input, which is critical for capturing intricate dependencies in drug–drug interaction (DDI) scenarios.

#### 2.2.2. Contrastive Learning

Contrastive learning is commonly implemented via the InfoNCE loss function, defined as

(2) $$L_{\mathrm{InfoNCE}}=-\log\left(\frac{\exp(\mathrm{sim}\left(q,k_{+}\right)/\tau )}{\exp\left(\mathrm{sim}\left(q,k_{+}\right)/\tau \right)+\sum_{i=0}^{K}\exp\left(\mathrm{sim}\left(q,k_{i}\right)/\tau \right)}\right)$$

Here, *q* denotes the anchor (or query) sample, $k_{+}$ is a positive (similar) sample, and each $k_{i}$ represents a negative (dissimilar) sample. The temperature parameter τ is a tunable hyperparameter that controls the sharpness of the similarity distribution. The similarity function sim(·,·) is typically implemented as cosine similarity. The principle behind Noise Contrastive Estimation (NCE) is to distinguish observed (positive) data from artificially generated noise (negatives) through a discriminative objective [33].

### 2.3. LSA-DDI Overview

**Figure 1:**
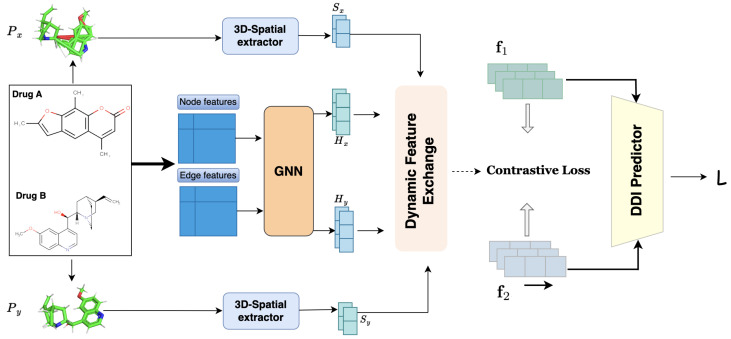
The figure illustrates the framework of a drug–drug-interaction prediction model. It includes the 3D spatial-feature extraction and GNN encoding processes for two drug molecules, A and B. Also covered are the dynamic feature-exchange mechanism, multiscale contrastive learning strategy, and final DDI predictor. The interactions between model components are detailed, showing the entire process of drug-interaction prediction via the integration of 3D spatial and topological features.

This study proposes a novel **Spatial-Contrastive-Attention-Based Drug–Drug-Interaction prediction framework** named **LSA-DDI**. As shown in Figure 1, the model integrates 3D molecular modeling, cross-modal dynamic feature fusion, and multiscale contrastive learning, effectively enhancing the prediction of stereochemistry-sensitive interactions. Figure 1 presents the four major modules of the LSA-DDI workflow: 3D spatial-feature extraction, 2D topological-graph encoding, dynamic feature exchange, and multiscale contrastive learning.

**Figure 2:**
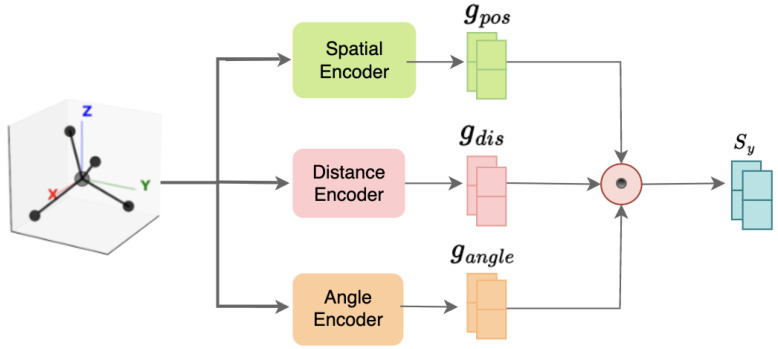
The structure of the 3D feature-extraction module, showing how spatial, distance, and angle encoders extract coordinate, distance, and angle information from molecular structures to generate a unified 3D spatial-feature representation.

In the 3D spatial feature extraction module, the SMILES representation of each drug is converted to a 3D molecular conformation using RDKit. Random spatial rotations are applied to improve the model’s robustness to conformational variance. Then, three parallel pathways extract atomic-level stereochemical features from coordinate, distance, and angular information. These features are fused using a dedicated fusion network to produce a unified 3D spatial representation, as illustrated in Figure 2.

For topological representation learning, LSA-DDI employs a multibranch GNN encoder composed of GAT and SAGPooling layers. These branches are designed to extract structural information at the atom, functional group, and molecular scaffold levels. The multiscale features are then fused via an MLP to generate the final 2D graph representation.

**Figure 3:**
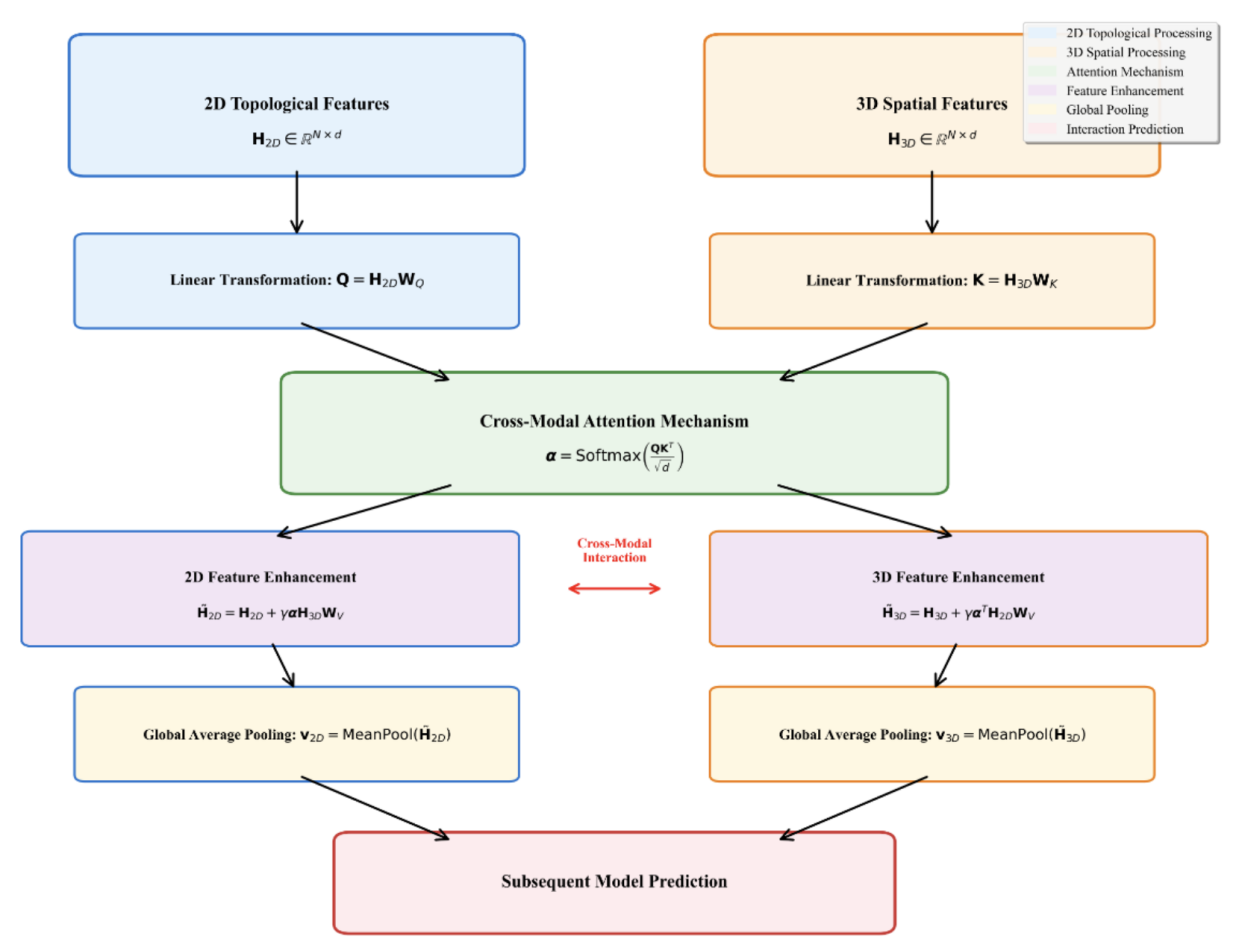
Dynamic Feature Exchange (DFE) mechanism. The mechanism uses linear transformations to generate query and key vectors, computes attention weights via a cross-modal attention mechanism, and enhances 2D and 3D features bidirectionally. Finally, it uses global average pooling to obtain feature representations for subsequent predictions.

To achieve deep semantic alignment between the 3D spatial and 2D topological modalities, LSA-DDI introduces a **Dynamic Feature Exchange (DFE)** mechanism. As shown in Figure 3, the DFE module adopts a bidirectional cross-attention strategy to dynamically regulate the information flow between modalities, enabling both 2D→3D and 3D→2D feature enhancement. This design improves the model’s ability to identify critical interaction regions between drug pairs.

Furthermore, LSA-DDI incorporates a **multiscale contrastive learning framework**. Drug representations at three structural levels—atomic, functional group, and scaffold—are used to perform InfoNCE-based contrastive learning. Scale-specific contrastive losses are adaptively weighted via learnable parameters and jointly optimized with the main task loss to promote better generalization. Finally, the DDI score is computed using **RESCAL tensor decomposition**, enabling relation-specific interaction prediction.

### 2.4. Three-Dimensional Feature Extraction

For each pair of drug molecules, $G_{x}$ and $G_{y}$, we first generate their 3D conformations from SMILES representations using the RDKit toolkit. To ensure robustness to conformational variations, we apply random rotation transformations by sampling Euler angles, θ∼U(0,2π), from a uniform distribution and constructing rotation matrices, $R_{\theta }$, to transform the initial atomic coordinates:

(3) $$X_{\mathrm{aug}}=R_{\theta }X_{\mathrm{init}}.$$

This rotational invariance augmentation ensures that the model learns the intrinsic structural features of molecules, independently of their absolute spacial orientations.

Our Spatial3DLayer captures molecular stereochemical information through three complementary feature-extraction pathways. First, the coordinate-feature encoder directly processes the augmented 3D coordinates, mapping the raw coordinates to a high-dimensional feature space through a multilayer perceptron structure:

(4) $$f_{\mathrm{pos}}=\mathrm{SpatialEncoder}\left(X_{\mathrm{aug}}\right),$$

where SpatialEncoder consists of linear layers, ReLU activation functions, layer normalization, and dropout, mapping 3D coordinates to a *d*-dimensional feature space. This encoder transforms atomic spatial-position information into latent representations through nonlinear transformations, enabling the model to learn associations between atomic positions and their chemical properties.

Subsequently, the distance feature encoder focuses on the molecule’s radial distribution features. First, we calculate the molecule’s geometric center, $c=\frac{1}{n}\sum_{i=1}^{n}X_{\mathrm{aug},i}$. Then, we compute the Euclidean distance of each atom to the center:

(5) $$d={∥X_{\mathrm{aug}}-c∥}_{2}.$$

These distance values are processed through the DistanceEncoder to obtain $f_{\mathrm{dist}}$, which maps one-dimensional distance features to a d/2-dimensional space. These radial features provide rotation-invariant information on molecular shape and atomic distribution, crucial for representing drug molecules’ structural characteristics.

Finally, we calculate normalized direction vectors:

(6) $$v_{\mathrm{norm}}=\frac{X_{\mathrm{aug}}-c}{{∥X_{\mathrm{aug}}-c∥}_{2}}.$$

These normalized vectors contain cosine values of the angles between atoms and coordinate axes, processed through AngleEncoder to obtain $f_{\mathrm{angle}}$, mapping 3D angular features to a d/2-dimensional space. Angular features provide information on atomic distribution in spherical coordinates, essential for understanding molecular spatial configuration and functional-group orientation.

Ultimately, we concatenate and fuse the three feature types:

(7) $$f_{\mathrm{combined}}=\left[f_{\mathrm{pos}};f_{\mathrm{dist}};f_{\mathrm{angle}}\right]$$

(8) $$s=\mathrm{FusionNetwork}(f_{\mathrm{combined}}),$$

where FusionNetwork employs linear transformations, ReLU activation functions, and layer normalization to map the 2d-dimensional concatenated features to a *d*-dimensional unified representation space. This multipathway feature extraction and fusion strategy enables our model to fully capture molecular 3D structural information, including absolute positions, relative distance distributions, and spatial orientations.

### 2.5. Dynamic Feature Exchange

To achieve efficient fusion of multimodal features, we design and introduce a DFE mechanism after extracting 2D topological-structure features and 3D spatial-structure features. This mechanism is used for semantic alignment and information enhancement between the two modalities.

Let the node-level representation output by the 2D encoder be $H_{2D}\in R^{N\times d}$, representing the atomic features of molecules in the topological structure. The output of the 3D encoder is $H_{3D}\in R^{N\times d}$, representing the geometric features in the spatial structure. Here, *N* denotes the number of atoms, and *d* is the feature dimension. The features of the two modalities will be fused in the DFE module.

To dynamically regulate cross-modal information, we employ an attention mechanism to generate gating weights. Specifically, query (Query) and key (Key) vectors are constructed through linear mappings:

(9) $$Q=H_{2D}W_{Q},\;K=H_{3D}W_{K},\;\alpha =\mathrm{Softmax}\left(\frac{QK^{⊤}}{\sqrt{d}}\right),$$

where $W_{Q},W_{K}\in R^{d\times d}$ are learnable parameters, and the attention matrix $\alpha \in R^{N\times N}$ represents the attention strength of each 2D atom for all 3D atoms.

After obtaining the attention weights, the features of the other modality are weighted and injected into the current modality to achieve bidirectional feature enhancement:

(10) $${\tilde{H}}_{2D}=H_{2D}+\gamma \;\cdot \;\alpha H_{3D}W_{V},\;{\tilde{H}}_{3D}=H_{3D}+\gamma \;\cdot \;\alpha^{⊤}H_{2D}W_{V},$$

where $W_{V}\in R^{d\times d}$ is the value projection matrix, and γ is a learnable scaling factor with an initial value of 0.1. This value helps to avoid instability caused by overly strong modality fusion during the early stages of training.

After the feature interaction is completed, global average pooling operations on ${\tilde{H}}_{2D}$ and ${\tilde{H}}_{3D}$ obtain independent vector representations for the two modalities:

(11) $$v_{2D}=\mathrm{MeanPool}\left({\tilde{H}}_{2D}\right),\;v_{3D}=\mathrm{MeanPool}\left({\tilde{H}}_{3D}\right).$$

These representations not only retain the unique structural information of each modality but also have the ability to respond to the other modality, which is beneficial for subsequent multiscale contrastive learning and interaction type recognition.

Finally, we combine multi-head outputs and predict interactions via RESCAL tensor decomposition:

(12) $$\mathrm{score}=\mathrm{Tr}({v_{2D}}^{T}W_{\mathrm{rel}}\left(r\right)v_{3D}).$$

where $W_{\mathrm{rel}}\left(r\right)\in R^{64\times 64}$ specifically encodes the *r*-th interaction type, enabling interpretable pattern learning. This architecture effectively captures the bidirectional, multiscale nature of DDI by leveraging both structural and spatial information.

### 2.6. Multiscale Contrastive Learning

To explore the implicit structural information within molecules, we implement a multiscale contrastive learning mechanism with drug representations from the bidirectional cross-attention layer as input, constructing contrastive constraints at different abstraction levels to encourage learning richer molecular interaction patterns. We select [1, 2, 4] as scale parameters, corresponding to the atomic, functional-group, and molecular-scaffold levels, based on the hierarchical molecule characteristics.

For drug pair representations $f_{1}$ and $f_{2}$, we perform scale-specific pooling operations:

(13) $$f_{1}^{s}=\mathrm{AvgPool}1d\left(f_{1},s\right),\;f_{2}^{s}=\mathrm{AvgPool}1d\left(f_{2},s\right).$$

We then apply L2 normalization for numerical stability:

(14) $${\hat{f}}_{1}^{s}=\frac{f_{1}^{s}}{\left|\right|f_{1}^{s}{\left|\right|}_{2}},\;{\hat{f}}_{2}^{s}=\frac{f_{2}^{s}}{\left|\right|f_{2}^{s}{\left|\right|}_{2}}.$$

The similarity matrix is computed using

(15) $$S^{s}=\frac{{\hat{f}}_{1}^{s}{\left({\hat{f}}_{2}^{s}\right)}^{T}}{\tau },$$

where τ=0.1−2.0 is a learnable hyperparameter (referred to as a ‘temperature parameter’ in machine learning, unrelated to physical temperature), which controls the distribution smoothness, with smaller values producing sharper distributions that enhance discriminative ability.

Our contrastive learning treats each drug pair and its 3D-augmented counterpart as positive pairs, while different drug pairs serve as negative samples. Specifically, for a batch of *N* drug pairs, we construct

- **Positive pairs:** ${\left\{\left(f_{i}^{s},f_{i}^{\prime s}\right)\right\}}_{i=1}^{N}$ for each scale *s*.
- **Negative pairs:** ${\left\{\left(f_{i}^{s},f_{j}^{s}\right),\left(f_{i}^{s},f_{j}^{\prime s}\right)\right\}}_{i\neq j}$ for each scale *s*.

The InfoNCE loss for scale *s* is defined as

(16) $$L_{\mathrm{contrast}}^{s}=-\frac{1}{N}\sum_{i=1}^{N}\log\left(\;\;\frac{\exp\left(\;\;\frac{\mathrm{sim}({\hat{f}}_{i}^{s},{\hat{f}}_{i}^{\prime s})}{\tau }\;\;\right)\;\;}{\exp\left(\;\;\frac{\mathrm{sim}({\hat{f}}_{i}^{s},{\hat{f}}_{i}^{\prime s})}{\tau }\;\;\right)\;\;+\sum_{j\neq i}\left[\;\;\exp\left(\;\;\frac{\mathrm{sim}({\hat{f}}_{i}^{s},{\hat{f}}_{j}^{s})}{\tau }\;\;\right)\;\;+\exp\left(\;\;\frac{\mathrm{sim}({\hat{f}}_{i}^{s},{\hat{f}}_{j}^{\prime s})}{\tau }\;\;\right)\;\;\right]}\;\;\right)\;.$$

where ${\hat{f}}_{i}^{s}$ and ${\hat{f}}_{i}^{\prime s}$ represent the $\ell_{2}$-normalized features of drug pair *i* and its 3D-augmented version at scale *s*, and τ is the temperature hyperparameter. The function sim(·,·) denotes cosine similarity.

To adaptively adjust scale importance, we introduce learnable weight parameters:

(17) $$w_{s}=\frac{\exp({\mathrm{ff}}_{s})}{\sum_{s^{\prime }\in \left\{1,2,4\right\}}\exp\left({\mathrm{ff}}_{s^{\prime }}\right)}.$$

The final multiscale contrastive loss combines scale-specific losses:

(18) $$L_{\mathrm{total}}=\sum_{s\in \{1,2,4\}}w_{s}L_{\mathrm{contrast}}^{s}$$

During training, we employ a dynamic weight adjustment strategy:

(19) $$\gamma =\gamma_{0}\;\cdot \;\left(1-\frac{t}{T}\right)$$

where $\gamma_{0}=0.1$ and T=500 epochs, *t* is the current epoch. This ensures an early focus on representation learning and task optimization later. The final loss combines the main task loss and contrastive learning loss:

(20) $$L=L_{\mathrm{main}}+\gamma \left(t\right)\;\cdot \;L_{\mathrm{total}}.$$

## 3. Experiments and Results

### 3.1. Dataset

To assess our method, we conducted experiments on a widely adopted real-world DDI benchmark, DrugBank [34]. This benchmark includes 191,808 drug pairs, involving 1706 distinct drugs and 86 interaction types. Each drug pair is associated with a single type of interaction that specifies how one drug influences the metabolism of the other.

We converted each drug’s SMILES into graph data using RDKit (version 2024.09.1) [18]. In this graph, the drug’s atoms were represented as nodes, while the chemical bonds formed the edges connecting these nodes, with corresponding features assigned to each node and edge. The detailed features of the atoms and bonds are shown in Table 1.

### 3.2. Experimental Setup

The SSI-DDI model comprises a 4-layer GAT with parameter sharing, with each layer having two multi-head attention heads (each head outputs 32-dimensional features, concatenated to 64 dimensions), followed by LayerNorm and ELU activation. The input data undergo preliminary LayerNorm processing, and each interaction type, $I_{r}$, corresponds to a learnable 64 × 64 relation matrix. For 3D-structure processing, molecular structures are generated from SMILES and augmented with random rotation. Coordinates, distances, and direction features are encoded via separate modules and fused through a linear layer for unified molecule representation. In feature fusion, the DFI module maps 2D features to Query and 3D features to Key, generating gating coefficients via attention weights for bidirectional feature injection (2D→3D and 3D→2D), using a learnable scaling factor, γ (initially 0.1). The final drug pair representation is generated via global average pooling. For training, multiscale contrastive learning (atomic, substructure, molecular levels) uses a three-scale InfoNCE loss, with dynamic loss weight combination via learnable parameters. The total loss combines contrastive and main task losses, the former decaying linearly with training epochs. Optimization uses AdamW (weight decay 1 × 10^−3^, batch size 512), with an initial learning rate of 5 × 10^−3^ and cosine annealing lr scheduling. We conducted experiments on an NVIDIA GeForce RTX 4090 GPU.

### 3.3. Baselines

We compared our method with the following baselines.

- MHCADDI [20]: Incorporates external message-passing protocols between drug structures, integrating joint drug information during the process of learning individual drug representations.
- SSI-DDI [26]: This model uses molecular graphs as inputs and employs GAT layers to extract substructure features.
- GMPNN-CS [35]: This gating mechanism enables the learning of substructures of diverse sizes and shapes from drug molecule graphs, with DDI event predictions derived from the interaction computations of these substructures.
- BDN-DDI [36]: A bidirectional dynamic network with a multihead attention mechanism designed to capture molecular-structure information and employed for adaptive feature extraction and dynamic message passing to predict DDIs.
- DGNN-DDI [37]: A directed message passing network incorporating a substructure attention mechanism, used to adaptively extract substructures and applied for DDI prediction.

### 3.4. Performance Evaluation of LSA-DDI in an Inductive Setting

Our initial experiments were conducted in a transductive setting. That is, the drugs in the test phase were also included in the training phase. To further evaluate our model’s generalization ability, we extended our experiments to a cold-start scenario involving completely unseen drugs. This setting better reflected real-world scenarios, where novel drugs must be predicted. To achieve this, we restructured our experimental protocol by splitting the dataset at the drug level rather than the interaction level.

Let $G_{\mathrm{new}}$ denote the set of new (unseen) drugs and $G_{\mathrm{old}}$ the set of seen drugs, satisfying

(21) $$G_{\mathrm{new}}\cup G_{\mathrm{old}}=G,\;G_{\mathrm{new}}\cap G_{\mathrm{old}}=\emptyset .$$

We then defined the splits as

(22) $$D_{\mathrm{train}}=\left\{\left(G_{x},G_{y},r\right)\in D|G_{x},G_{y}\in G_{\mathrm{old}}\right\},$$

(23) $$D_{S1}=\left\{\left(G_{x},G_{y},r\right)\in D|G_{x},G_{y}\in G_{\mathrm{new}}\right\},$$

(24) $$\begin{array}{cc} D_{S2}=\{\left(G_{x},G_{y},r\right)\in D\;|\; & \left(G_{x}\in G_{\mathrm{old}}\wedge G_{y}\in G_{\mathrm{new}}\right)\\ \; & \;\;\;\;\vee \\ \; & (G_{x}\in G_{\mathrm{new}}\wedge G_{y}\in G_{\mathrm{old}})\}. \end{array}$$

$D_{\mathrm{train}}$ contains DDI tuples of drug pairs (known drugs) used for training, while $D_{S1}$ comprises tuples of unknown drugs not present in $D_{\mathrm{train}}$. The remaining tuples (where one drug belongs to $D_{S1}$ and the other to $D_{\mathrm{train}}$) form $D_{S2}$. The model was trained on $D_{\mathrm{train}}$ and evaluated on both $D_{S1}$ and $D_{S2}$.

**Figure 4:**
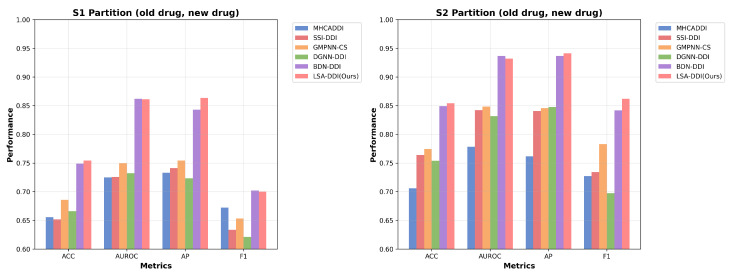
Figure shows the performance evaluation of different models on the S1 and S2 partitions (involving old and new drug combinations). By comparing the models’ performance across ACC, AUROC, AP, and F1 metrics, it visually demonstrates the performance of the LSA-DDI model in multiple scenarios.

Table 2 compares our method with baseline approaches using the same model configuration as in Section IV-B, but with a cosine-annealing learning rate schedule (initial learning rate = 5 × 10^−3^) and a new drug ratio of 1/5. Based on the average and standard deviation of three runs reported in Table 2, our LSA-DDI method achieved the highest AUROC on known–unknown drug pairs, significantly outperforming existing methods. Table 3 presents the detailed comparison in cold-start scenarios, while all methods experienced a drop in performance, ours still achieved a competitive AUROC of 86.12%, While BDN-DDI demonstrated comparable performance in cold-start scenarios (AUROC: 86.20% in S1, 93.68% in S2), LSA-DDI showed consistent advantages through its systematic 3D spatial feature extraction and dynamic cross-modal attention mechanisms. The performance improvements, though modest in absolute terms, reflect the effectiveness of incorporating stereochemical information for DDI prediction. This can also be seen from Figure 4. This strategy generated diverse spatial representations, enhancing model generalization by leveraging common substructures and spatial features across different drugs. Our model consistently outperformed the other methods in most performance metrics, showing the highest median values and tightest interquartile ranges.

### 3.5. Ablation Experiment

To delve deeper into the factors that bolstered our model’s generalization, we ran ablation experiments on test data from S1 and S2 partitions by methodically removing each component of the LSA-DDI framework and observing the subsequent performance shifts. Using three-fold cross validation, we assessed these modified versions against our complete LSA-DDI model.

- **wo_3d**: Model lacking 3D information. We explored how eliminating 3D stereochemical data of drugs affected drug-interaction modeling.
- **wo_DFE**: Model without the dynamic feature exchange mechanism. We assessed the dynamic feature exchange mechanism’s role in capturing interaction patterns between drugs.
- **wo_constrast**: Model lacking multiscale contrastive learning. We examined how multiscale contrastive learning contributed to capturing multiscale structural features.
- **wo_3D_constract**: Model without both 3D data augmentation and multiscale contrastive learning. We investigated the combined effect of spatial information and contrastive learning.

**Figure 5:**
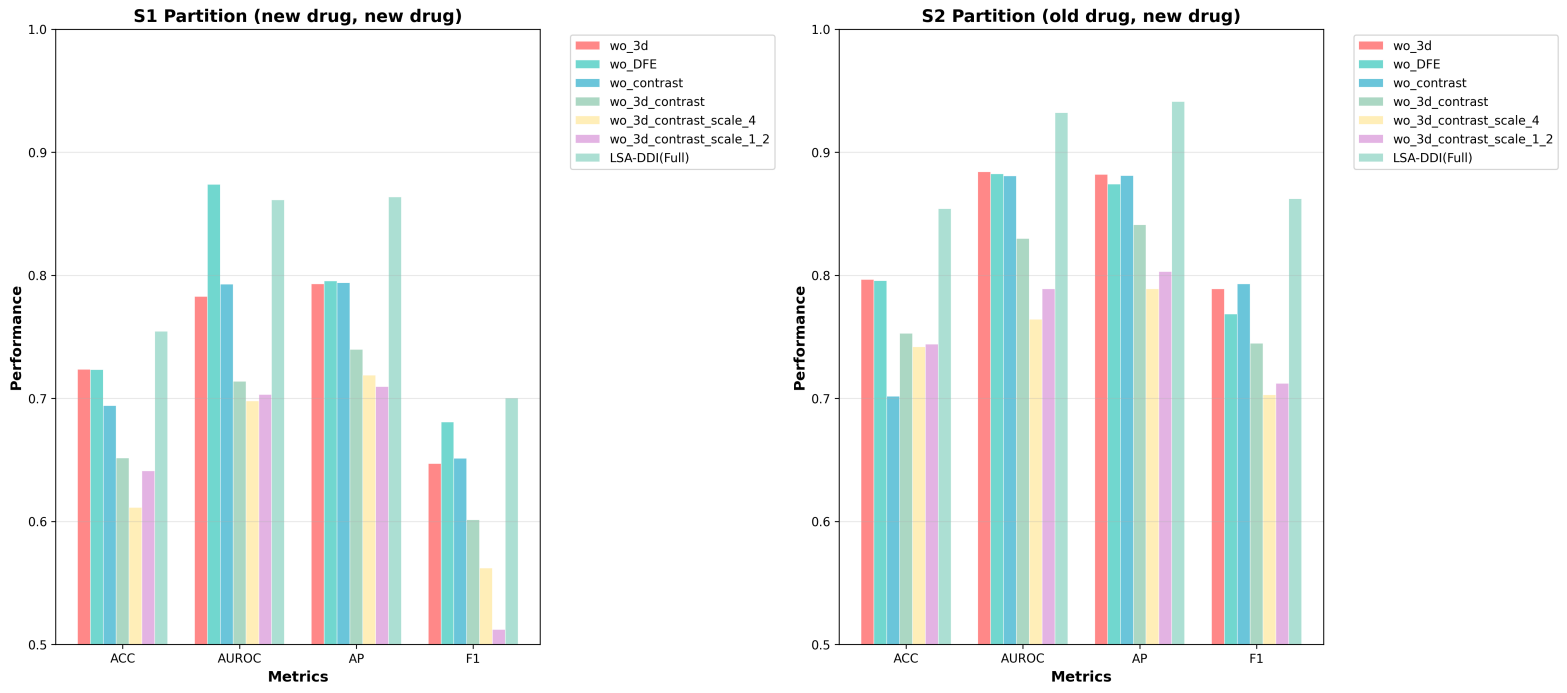
Figure presents the results of an ablation study, comparing the performance of the LSA-DDI model and its variants across S1 and S2 partitions (new drug–new drug and old drug–new drug combinations, respectively).

Table 4 shows that LSA-DDI outperformed all its variants, confirming the effectiveness of each component. Removing the 3D module (wo_3D) significantly reduced performance, demonstrating that incorporating spatial geometric information effectively captures chemical features and addresses the cold-start problem. Similarly, the results from wo_DFE indicate the crucial role of bidirectional cross-attention in identifying key drug regions and aligning features, while wo_contrast confirms that contrastive learning enhanced the feature discriminability and preserved molecular topology invariance. In the wo_3D_contrast experiment, with neither 3D information extraction nor contrastive learning, the model depended only on 2D topological information, leading to a significant performance decline. To test the effectiveness of the scale parameters 1, 2, 4, we conducted ablation studies involving removing or keeping only molecule-level features (scale = 4), both of which caused sharp drops (Table 4). This shows the need for multiple scales. It underlines how crucial it is to combine information from various structural spaces and to use their combined effects for accurate DDI prediction. This can also be clearly seen in Figure 5.

### 3.6. Case Study

**Figure 6:**
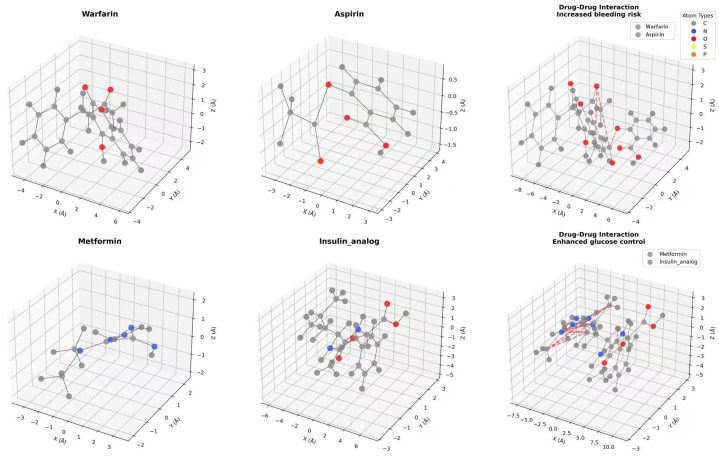
Three-dimensional data augmentation case study.

Figure 6 presents the spatial visualizations of the 3D molecular structures and DDIs processed by LSA-DDI. The red dashed lines in the visualizations represent critical interaction bonds and key stereochemical sites identified by the model’s attention mechanism as crucial for DDIs. In the Warfarin–aspirin case, the relative positions of Warfarin’s coumarin structure and aspirin’s salicylic acid structure in 3D space determine their synergistic anticoagulant effect, with the red dashed lines highlighting the specific interaction sites where these structures achieve optimal spatial complementarity. LSA-DDI successfully identified the interaction pattern of increased bleeding risk through extraction of coordinate, distance, and angle features at these critical sites. The metformin–insulin analog case further validated the model’s adaptability in handling different pharmacological mechanisms, where the red dashed lines indicate the complementary interaction regions between metformin’s biguanide structure and insulin’s polypeptide structure that contribute to their synergistic glucose-lowering effect. These visualization results fully demonstrate that LSA-DDI can accurately capture stereochemical information and spatial interaction patterns that traditional 2D methods cannot handle, providing important technical support for predicting complex DDIs.

**Figure 7:**
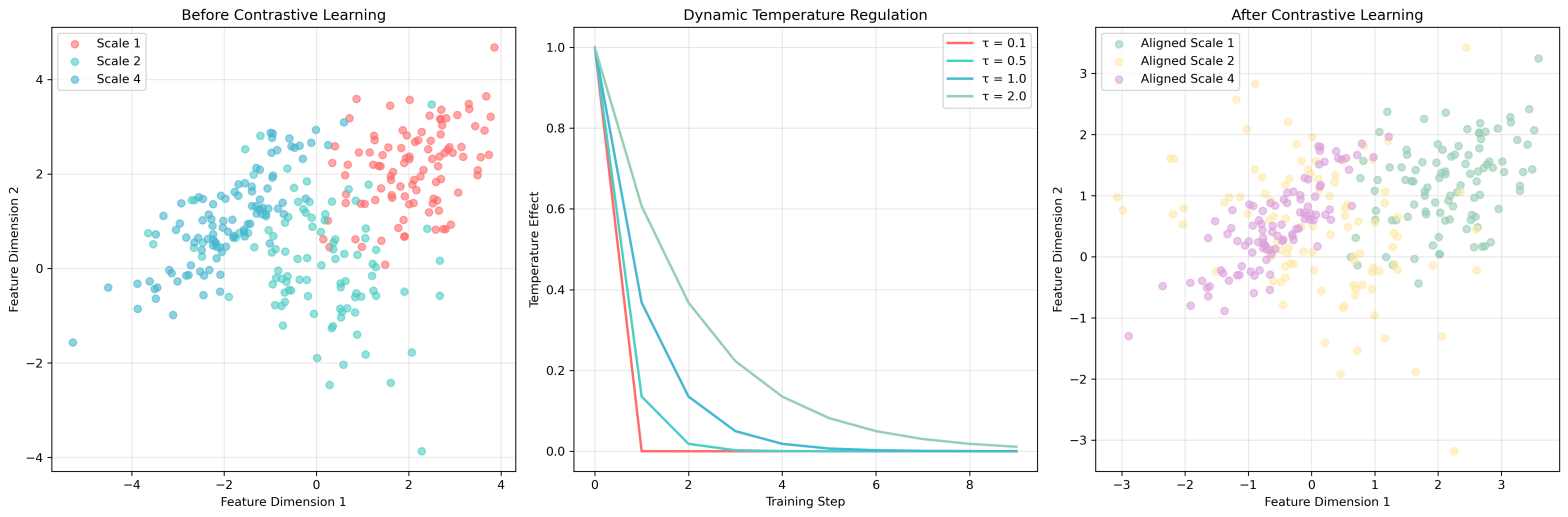
Comparative study case analysis.

As shown in Figure 7, the DFE mechanism achieved dynamic fusion of 2D topological features and 3D spatial features through bidirectional attention. The upper left plot shows that the original features exhibited obvious modal differences, while the upper right plot demonstrates the bidirectional information flow of 2D→3D and 3D→2D attention weights. The features enhanced after processing (lower left plot) achieved better semantic alignment, with feature similarity significantly improving from 0.03 to 0.95, demonstrating the effectiveness of the DFE mechanism.

**Figure 8:**
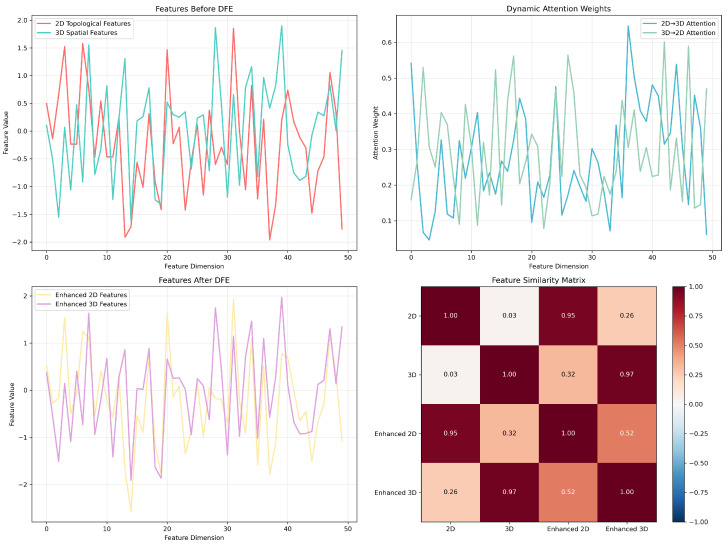
DFE data fusion case analysis.

The multiscale contrastive learning framework in Figure 8 demonstrates the working principle of the dynamic temperature regulation mechanism. Before contrastive learning, features at different scales (Scale 1, 2, 4) exhibited scattered distributions, while dynamic temperature parameters (τ=0.1–2.0) adjusted learning intensity during training, ultimately achieving feature alignment and clustering optimization. This mechanism enabled LSA-DDI to simultaneously learn effective feature representations at multiple levels, demonstrating excellent generalization performance when handling new drug combinations.

## 4. Conclusions

We proposed a novel drug–drug interaction prediction model, LSA-DDI, which integrates three key innovations to enhance prediction accuracy. Through a 3D spatial-feature extractor and position information embedding, the model can accurately capture and retain the fine stereochemical characteristics of drug molecules, use a bidirectional cross-attention module to accurately identify critical interaction regions between drugs, and implement a multiscale contrastive learning framework with dynamic InfoNCE loss to effectively align 2D and 3D features, strengthening the model’s consistency and generalization capability. In benchmark tests on DrugBank, LSA-DDI achieved competitive performance and demonstrated consistent improvements over baseline methods. From a clinical perspective, the model can not only support new drug development and polypharmacy safety assessment, but its interpretable attention patterns also provide deep pharmacological insights into interaction mechanisms, offering robust support for precision medical decision making.

Moreover, we are aware of the potential ethical issues that may arise from the practical application of such models. The predictions generated by LSA-DDI should be used solely for clinical-decision support, with final treatment decisions made by qualified medical professionals. During the development and deployment of the model, it is essential to strictly protect patient privacy and ensure the legal and compliant use of all data. Finally, the model’s performance and fairness across different populations and clinical settings should be continuously monitored and improved.

## Figures and Tables

**Table 1:** Chemical feature overview.

Feature	Type	Dimension
**Node Features**		
The atomic symbol	Node	44
The degree of the atom	Node	1
The implicit valence	Node	1
The formal charge	Node	1
The number of radical electrons	Node	1
The hybridization	Node	5
Whether or not the atom is aromatic	Node	1
The total number of hydrogens on the atom	Node	1
Subtotal for Node Features		55
**Bond Features**		
Bond type	Bond	4
Conjugation	Bond	1
In ring	Bond	1
Subtotal for Bond Features		6
**Total Features**		61

**Table 2:** The performance of each model in the warm-start task. The maximum value of each column is bolded.

Model	ACC	AUROC	AP	F1
MHCADDI	82.60	88.40	89.12	85.40
SSI-DDI	95.48	97.49	97.86	96.07
GMPNN-CS	94.89	97.52	97.48	95.30
DGNN-DDI	96.32	98.20	98.22	95.23
BDN-DDI	96.42	98.28	98.24	96.30
LSA-DDI(Ours)	**97.43**	**98.70**	**98.49**	**96.50**

**Table 3:** The performance of each model in the cold-start tasks. The maximum value of each column is bolded.

Model	S1 Partition (New Drug, New Drug)				S2 Partition (Old Drug, New Drug)			
	ACC	AUROC	AP	F1	ACC	AUROC	AP	F1
MHCADDI	65.53	72.48	73.29	67.21	70.58	77.84	76.15	72.73
SSI-DDI	65.18	72.58	74.10	63.32	76.38	84.22	84.08	73.42
GMPNN-CS	68.57	74.96	75.44	65.32	77.45	84.84	84.56	78.29
DGNN-DDI	66.57	73.21	72.32	62.11	75.39	83.15	84.75	69.71
BDN-DDI	74.88	**86.20**	84.32	70.21	84.92	**93.68**	93.64	84.18
LSA-DDI (Ours)	**75.45**	86.12	**86.37**	**70.02**	**85.41**	93.21	**94.12**	**86.21**

**Table 4:** Performance demonstration of LSA-DDI and its variants on S1 and S2 partitions. The maximum value of each column is bolded.

Model	S1 Partition (New Drug, New Drug)				S2 Partition (Old Drug, New Drug)			
	ACC	AUROC	AP	F1	ACC	AUROC	AP	F1
wo_3d	72.35	78.28	79.30	64.70	79.65	88.40	88.20	78.90
wo_DFE	72.33	87.38	79.53	68.07	79.55	88.23	87.42	76.86
wo_contrast	69.41	79.27	79.40	65.12	70.18	88.07	88.10	79.29
wo_3d_contrast	65.14	71.37	73.97	60.13	75.29	82.98	84.10	74.47
wo_3d_contrast_scale_4	61.14	69.80	71.88	56.21	74.19	76.43	78.90	70.29
wo_3d_contrast_scale_1,2	64.11	70.31	70.96	51.22	74.39	78.90	80.30	71.22
LSA-DDI (Ours)	**75.45**	**86.12**	**86.37**	**70.02**	**85.41**	**93.21**	**94.12**	**86.21**

## Data Availability

The data supporting the reported results are publicly available. This study utilized the DrugBank database (version 4.0), which contains 191,808 drug pairs involving 1706 distinct drugs and 86 interaction types. The dataset can be accessed at https://go.drugbank.com/. No new datasets were generated during this study. All experimental configurations and hyperparameters are detailed in the Methods section to ensure reproducibility.
